# Early morning off in patients with Parkinson’s disease: a Chinese nationwide study and a 7-question screening scale

**DOI:** 10.1186/s40035-020-00208-z

**Published:** 2020-07-06

**Authors:** Chao Han, Wei Mao, Jing An, Lifei Jiao, Piu Chan, on behalf of the China Early Morning Off (EMO) Study Group

**Affiliations:** 1 National Clinical Research Center for Geriatric Disorders, Xuanwu Hospital of Capital Medical University, Beijing, China; 2 Department of Neurobiology, Neurology and Geriatrics, Xuanwu Hospital of Capital Medical University, 45 Changchun Road, Beijing, 100053 China; 3 Beijing Institute of Geriatrics, Xuanwu Hospital of Capital Medical University, 45 Changchun Road, Beijing, 100053 China; 4 Clinical Center for Parkinson’s Disease, Xuanwu Hospital of Capital Medical University, 45 Changchun Road, Beijing, 100053 China; 5 Key Laboratories for Neurodegenerative Diseases of the Ministry of Education, Xuanwu Hospital of Capital Medical University, 45 Changchun Road, Beijing, 100053 China; 6 Beijing Key Laboratory for Parkinson’s Disease, Xuanwu Hospital of Capital Medical University, 45 Changchun Road, Beijing, 100053 China; 7 Parkinson Disease Center of Beijing Institute for Brain Disorders, Xuanwu Hospital of Capital Medical University, 45 Changchun Road, Beijing, 100053 China; 8 Advanced Innovative Center for Human Brain Protection, Xuanwu Hospital of Capital Medical University, 45 Changchun Road, Beijing, 100053 China; 9 Medical department, Lundbeck (Beijing) Pharmaceutical Co. ltd, Beijing, China

**Keywords:** Parkinson’s disease, Early morning off, Motor symptoms, Non-motor symptoms, Screening scale

## Abstract

**Background:**

Early morning off (EMO) is a common feature of Parkinson’s disease (PD). This study aimed to characterize its clinical features and develop a convenient and pragmatic self-assessment instrument in a Chinese nationwide population.

**Methods:**

This study was conducted on 942 PD patients admitted to 55 clinic centers for movement disorders between June 2018 and May 2019 in China. Stepwise logistic regression analyses were performed to determine potential risk factors and the most predictive symptoms of EMO, as well as whether EMO was an independent risk factor of functional dependency in daily life. Based on this, a 7-question scale was derived for EMO screening. Diagnostic accuracy of this scale was assessed from the area under the receiver operative characteristic curve (AUROC) and its 95% confidence intervals (CIs). We further calculated sensitivity, specificity, positive predictive value (PPV), and negative predictive value (NPV) for the optimal cutoff point.

**Results:**

EMO occurred in 49.2% of PD patients across all disease stages. We identified 7 symptoms most predictive of EMO, including bradykinesia or rigidity, excessive sweating or salivation, difficulty in turning on or getting out of bed, muscle cramp, fatigue or sleepiness, frozen state or freezing gait, and tremor. The resulting 7-item scale was confirmed to be of good discrimination with a relatively large AUROC of 0.83, a relatively high sensitivity of 75.7%, specificity of 77.5%, PPV of 76.5%, and NPV of 76.7%. Nonideal nighttime sleep, long PD duration, advanced H&Y stages, posture instability gait difficulty-dominant or mixed subtypes, and high levodopa dose were independently associated with increased risk of EMO. EMO patients were at 87% higher (OR = 1.87, 95%CI: 1.07–3.32) risk of experiencing functional dependency in daily living compared with their counterparts.

**Conclusions:**

We demonstrated that EMO is a common feature for PD patients across all disease stages and put forward an EMO-specific screening card of sufficient accuracy and brevity. Meanwhile we have thrown some light upon potential determinants and negative health effects of EMO. Our findings may exert great impact on improving the awareness, recognition and management of EMO in PD patients.

## Background

Parkinson’s disease (PD) is the most common movement disorder and the second most common neurodegenerative disease only next to Alzheimer’s disease [1, 2]. The past three decades have witnessed an alarming increase in the total number of PD patients from 2.5 million in 1990 to 6.1 million in 2016 worldwide [3]. PD was also recognized as one of the leading causes of disability with a global report estimating that this devastating disease has caused 3.2 million disability-adjusted life-years and 0.2 million deaths in 2016 [3]. With the aging population and prolonging life expectancy, it was bound to impose heavier medical and economic burden in the coming years.

As we all know, most PD patients have to receive levodopa treatment and many of them will eventually develop a range of levodopa-induced complications by 15 years [4, 5]. Generally, the initial clinical complication is a predictable end-of-dose deterioration in therapeutic benefit, commonly termed as wearing-off and characterized by a wide range of motor (MS) and non-motor symptoms (NMS) [6]. At present, extensive studies have documented the epidemiology, prediction, and management of daytime wearing-off [6–13]. By contrast, far less information can be obtained regarding this issue for early morning period as yet, among which early morning akinesia received relatively more attention [14–21]. These studies have provided a general profile of early morning off (EMO) and proposed several promising drugs in decreasing or improving morning fluctuations. Whereas, available data were mainly based on single-center observational studies or clinical trials conducted among Caucasian populations. There is a paucity of evidence from representative multicenter studies among Asian population, especially from China. It is well accepted that the pattern at start of a day often dictates how the patient will feel during the rest of daytime and represents a critical moment to adjust his or her medication use [14, 22]. EMO also has a potential to impact the quality of life (QOL) for both patients and care givers, and influence treatment decisions [14, 15, 23]. However, lack of awareness, limited consultation time, and insufficient communication between clinicians and patients often preclude timely recognition of EMO in routine clinical visits. Therefore, it is imperative to construct a convenient and pragmatic screening tool for EMO at present.

The main aims of our study are to characterize the clinical features of EMO, including the spectrum of cardinal symptoms and potential determinants, and to develop a self-administered screening tool consisting of symptoms most predictive of EMO and of sufficient accuracy and brevity to be applied in clinical practice. Meanwhile we attempt to verify whether EMO is an independent prognostic factor for functional dependency in their daily living. In summary, this study is expected to provide a comprehensive understanding of EMO phenomenon, including its prevalence, clinical manifestations, risk factors, brief screening, and adverse health effects.

## MATERIALS and METHODS

### Study participants

This is nationwide multicenter registry study (http://www.chictr.org.cn/index.aspx, China Trial Register Number: ChiCTR1800019264). Our study consecutively enrolled 1002 outpatients who were admitted to 55 clinic centers for movement disorders selected from the Chinese Parkinson Alliance between June 2018 and May 2019. Qualified participants met the following criteria: (1) with a confirmed diagnosis of idiopathic PD according to the 2015 Movement Disorders Society criteria; (2) aged ≥30 years old; (3) with a PD duration of no less than one year; (4) under prescribing treatment of a steady dose of dopaminergic drugs (including levodopa and dopamine agonists (DA), except for monoamine oxidase type B (MAO-B) inhibitors) for at least 30 days before enrollment. Specifically ruling out MAO-B inhibitor treatment from this study was to allow for a predesigned delivery of this drug to EMO subgroups in subsequent phase and therefore investigate its independent effect on EMO. Another reason was that rasagiline, a common-used and long-acting MAO-B inhibitor, might decrease the occurrence of EMO and relieve related symptoms during OFF period as reported elsewhere [17, 24, 25]; (5) able and willing to participate the survey with a series of scales and questionnaires; (6) agreed to provide informed consent. Participants were excluded if they: (1) were atypical or secondary PD patients; (2) were accompanied with serious internal or surgical complications, including cardiovascular disease, respiratory disease, endocrine disease, urological disease, metabolic disease, malignant tumor, etc.; (3) ever underwent neurosurgical treatment, including pallidotomy, thalamotomy, deep brain stimulation or organ transplantation; (4) with a Mini-Mental State Examination (MMSE) score < 24. Ethical approvals were obtained from respective institutional ethical committees.

### Information collection

The collection of baseline information was carried out by trained movement disorder specialists during routine clinic visits. Sociodemographic characteristics (sex, age, education level, occupation), family history of PD, age of PD onset, medical history, treatment history, and recent sleep qualities were recorded using a standardized questionnaire. Sleep quality was measured by self-evaluation on overall nighttime sleep (good/general/poor) and number of nocturnal arousals (none/1–5 times/≥5 times) in the past week. Height and weight were measured with participants wearing light clothes and bare foot. Body mass index (BMI) was calculated as kilograms per meters squared (kg/m^2^). Clinical assessments included MMSE, clinical type of PD, symmetry of symptoms, Hoehn-Yahr (H&Y) stage, and Schwab-England Activities of Daily Living Scale (SES). The levodopa equivalent daily dosage (LEDD) was also calculated for each participant. Daily functional dependency was defined as a SES score less than 80% (SES ≥ 80% implies completely independent in most chores, SES < 80% implies not completely independent).

The diagnosis of EMO was also made by experienced PD specialists in this study. Since there was no established definition of EMO in PD patients, the presence of EMO was identified using the empiric diagnostic criteria as being in “off” state on morning waking, while related symptoms could be alleviated after taking the first dosage of dopaminergic drug. To detect the most predominant warning symptoms associated with EMO, we further constructed a questionnaire involving 15 specific questions on common MS and NMS to be completed by the participants. The questionnaire covered 7 motor domains and 8 non-motor domains modified from the Movement Disorders Society Unified Parkinson Disease Rating Scale (MDS-UPDRS) and 30-item NMS questionnaire (NMSQ-30). In this survey, participants ticked whether they have suffered from the following symptoms after waking up and before the first dosage of antiparkinsonian drug in the morning during the past week: (1) tremor of limbs or lip area; (2) muscle cramp; (3) difficulty in turning on or getting out of bed; (4) bradykinesia or rigidity; (5) frozen state or freezing gait; (6) dysphagia; (7) difficulty in washing or dressing; (8) pain that affects sleep posture (upper or lower limbs) forces awake early in the morning; (9) fatigue or sleepiness; (10) nocturnal frequent urination or urinary urgency; (11) in low mood or depression; (12) excessive sweating or salivation; (13) dizziness; (14) irritability or restlessness; (15) anxiety. The presence of each symptom was ascertained if it was improved or disappeared after a morning scheduled dosage of dopaminergic drug (Table S1).

Among the 1002 participants who met inclusion criteria and completed required surveys, 60 were subsequently excluded due to incomplete information on EMO status, leaving 942 subjects involved in the final analyses.

### Statistical analyses

Baseline characteristics were summarized based on EMO status. Continuous variables were described as mean ± standard deviation (SD) and compared by *t* test, while categorical variables were described as percentages and compared by *Chi-square* test. Binomial distribution was used to estimate point prevalence with 95% confidence intervals (CIs) of EMO across difference H&Y stages. We further calculated and ranked the proportions of 15 common MS and NMS that occurred during EMO period to identify the typical clinical manifestations of EMO. To detect potential risk factors of EMO, we calculated odds ratios (ORs) and corresponding 95% CIs of the baseline characteristics using both univariate, age- and sex-adjusted, and multivariate binary stepwise logistic regression models. The stepwise model selected variables based on a strategy using Akaike information criteria (AIC) which balanced goodness of fit with parsimony of predictors.

In addition, a separate stepwise logistic regression analysis was performed to identify the most predominant symptoms of EMO within the given 15 MS and NMS. Based on this, we derived a simple EMO screening scale, which contained all significant domains retaining in the model with one point assigned to each domain according to the presence of corresponding symptom. Stepwise logistic regression model was also used to investigate whether EMO was an independent risk factor of functional dependency in daily life.

We assessed the performance of derived EMO scale using tests for discrimination and calibration. We used the area under the receiver operative characteristic curve (AUROC) and its 95% CI to evaluate discrimination, and further tested whether it could be substantially improved by adding formerly imputed independent risk factors of EMO. We also assessed calibration that compares the observed proportions of EMO versus controls within equally sized groups categorized by their predicted probability. Hosmer-Lemeshow goodness of fit statistic was used to determine how well the average predicted probability within specified groups matches the proportion observed. Diagnostic accuracy of this scale was assessed and internally validated using bootstrapping methodology with 1000 repetitions to limit the effect of optimistic bias. Youden index (J) was used to identify the optimal cutoff point at which both sensitivity and specificity are maximized. We further calculated sensitivity, specificity, positive predictive value (PPV) and negative predictive value (NPV) for the cutoff point.

All statistical analyses were performed using the R software (version 3.5.1; R Development Core Team 2018, www.R-project.org). In particular, analyzing and comparison of ROC curves were performed using the pROC package and Delong test [26]. Two-tailed *P* values < 0.05 were considered statistically significant.

## Results

**Fig. 1:**
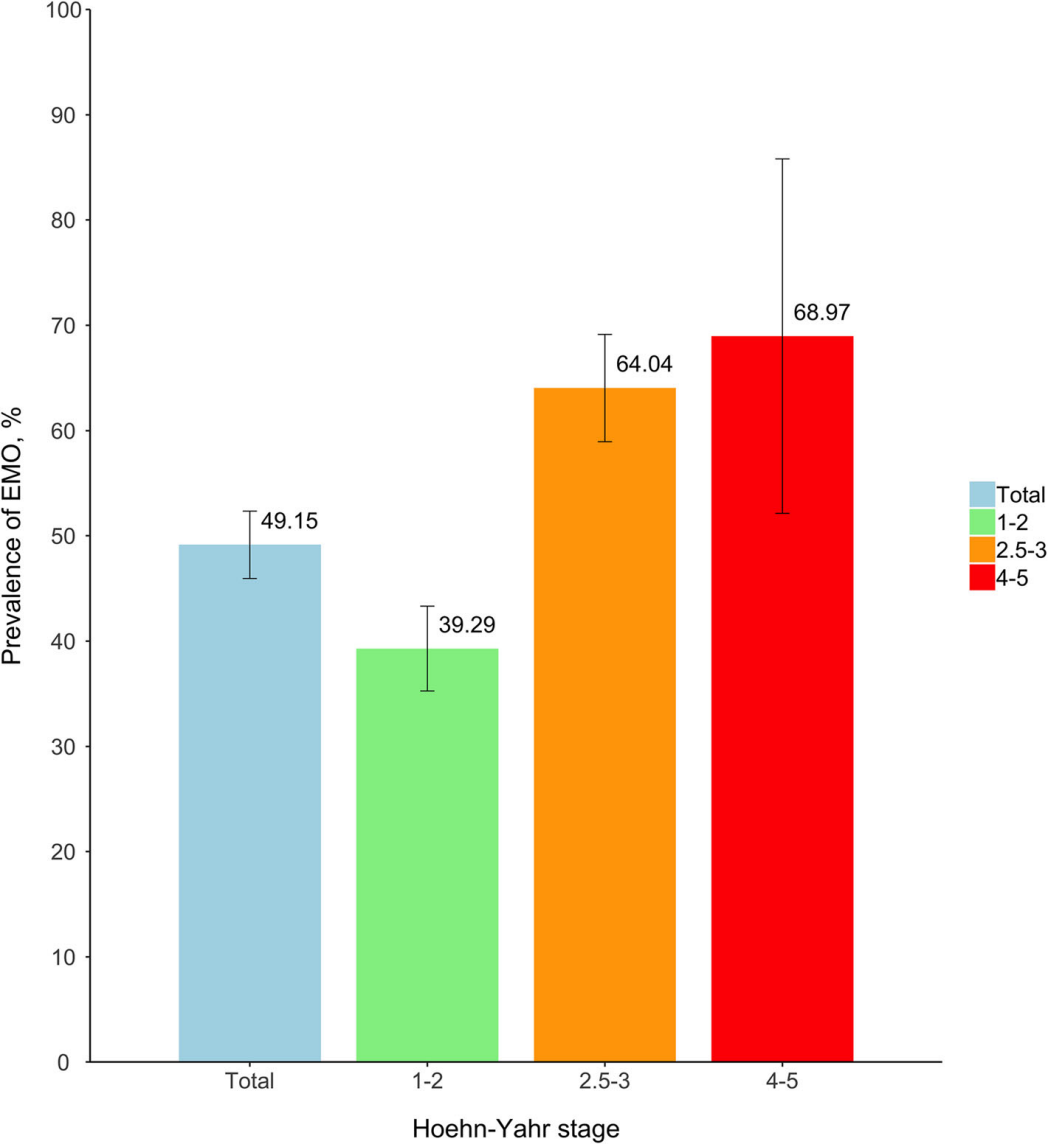
Prevalence of EMO in the participants at different stages of PD.

Our study consecutively included 942 PD patients with an average disease duration of 5.7 years (range: 1–32 years), of whom 48.1% were women and the mean age was 64.0 years. Demographic and clinical characteristics are shown in Table 1, overall and by EMO status. In total, EMO was diagnosed in 463 patients [49.2% (46.0–52.3%)] with a mean disease duration of 6.5 years and H&Y stage of 2.3. It was prevalent across all disease stages and showed an apparent upward trend with increasing stages (Fig. 1). EMO patients were more likely to wake up frequently at night, have a poorer nighttime sleep, longer PD duration, more advanced H&Y stages, and higher LEDD. Comparing with tremor-dominant (TD) patients, posture instability gait difficulty-dominant (PIGD) and mixed groups were more susceptible to experience EMO period. Besides, EMO patients generally suffered from more MS and NMS than their counterparts, leading to greater dependency in their daily life (Table 1 and Table S2). Analogously, both univariate and age- and sex-adjusted regression analyses proved that nonideal nighttime sleep, frequent nocturnal arousals, long PD duration, advanced H&Y stages, PIGD and mixed subtypes, and higher levodopa dose were significantly associated with increased EMO risk (all *P* < 0.05, Table 2). These factors were consistently retained in the final multiple stepwise logistic regression model except for nocturnal arousals. The relative risk of EMO was 2.08 (95%CI: 1.41–3.07) and 3.29 (95%CI: 2.14–5.08) for general and poor nighttime sleep, respectively; 1.78 (95%CI: 1.24–2.57) and 1.29 (95%CI: 0.48–3.64) for moderate and severe H&Y stage, respectively; 1.72 (95%CI: 1.16–2.55) and 1.78 (95%CI: 1.17–2.71) for PIGD and mixed patients, respectively; 1.06 (95%CI: 1.02–1.11) per year increase in PD duration; and 1.08 (95%CI: 1.01–1.16) per 100mg increment in LEDD. In addition, 65–74 years age group was at 79% (OR = 1.79, 95%CI: 1.09–2.96) higher risk of EMO compared to those aged < 55 years. Failure to reach statistical significance in the association for severe H&Y stage might largely attribute to relatively small sample size in this subgroup.

**Table 1:** Demographic and clinical characteristics of the study participants.

Baseline variables	Overall (*n* = 942)	EMO patients (*n* = 463)	Non-EMO patients (*n* = 479)	P Value
Age, y	64.0 ± 9.3	64.4 ± 8.8	63.5 ± 9.7	0.142
Age group				
< 55 years	149 (15.8)	63 (13.6)	86 (18.0)	0.269
55–64 years	334 (35.5)	166 (35.9)	168 (35.1)	
65–74 years	329 (34.9)	171 (36.9)	158 (33.0)	
> =75 years	130 (13.8)	63 (13.6)	67 (14.0)	
Sex				
Male	489 (51.9)	244 (52.7)	245 (51.1)	0.681
Female	453 (48.1)	219 (47.3)	234 (48.9)	
BMI groups				
Normal	550 (59.1)	278 (61.0)	272 (57.3)	0.279
Overweight or obese	381 (40.9)	178 (39.0)	203 (42.7)	
Education level				
Primary school or lower	223 (24.3)	106 (23.6)	117 (24.9)	0.834
Middle or high school	520 (56.6)	259 (57.6)	261 (55.7)	
University or higher	176 (19.2)	85 (18.9)	91 (19.4)	
Occupation				
Unemployed	84 (9.1)	46 (10.2)	38 (8.1)	0.192
Worker or farmer	363 (39.4)	166 (36.6)	197 (42.1)	
Professional technician or others	474 (51.5)	241 (53.2)	233 (49.8)	
Family history of PD				
No	847 (90.6)	416 (90.6)	431 (90.5)	1.000
Yes	88 (9.4)	43 (9.4)	45 (9.5)	
Overall nighttime sleep				
Good	331 (35.4)	113 (24.6)	218 (45.8)	< 0.001
General	342 (36.5)	181 (39.3)	161 (33.8)	
Poor	263 (28.1)	166 (36.1)	97 (20.4)	
Nocturnal arousals				
None	83 (8.9)	30 (6.5)	53 (11.2)	0.003
1–5 times	785 (84.0)	387 (84.1)	398 (83.8)	
> 5 times	67 (7.2)	43 (9.3)	24 (5.1)	
Age of PD onset, y	58.3 ± 9.9	57.9 ± 9.3	58.6 ± 10.5	0.302
PD duration	5.7 ± 4.3	6.5 ± 4.4	5.0 ± 3.9	< 0.001
H&Y stages	2.1 ± 0.7	2.3 ± 0.7	2.0 ± 0.7	< 0.001
H&Y group				
Mild (stage of 1–2)	560 (60.2)	220 (47.9)	340 (72.0)	< 0.001
Moderate (stage of 2.5–3)	342 (36.7)	219 (47.7)	123 (26.1)	
Severe (stage of 4–5)	29 (3.1)	20 (4.4)	9 (1.9)	
Symmetry of symptoms				
No	854 (93.2)	421 (94.4)	433 (92.1)	0.217
Yes	62 (6.8)	25 (5.6)	37 (7.9)	
Clinical type				
TD	404 (44.7)	166 (37.5)	238 (51.7)	< 0.001
PIGD	268 (29.7)	142 (32.1)	126 (27.4)	
Mixed	231 (25.6)	135 (30.5)	96 (20.9)	
Medication use				
Levodopa monotherapy	143 (18.7)	59 (15.9)	84 (21.4)	0.146
Levodopa + DA	374 (49.0)	186 (50.1)	188 (47.8)	
Others	247 (32.3)	126 (34.0)	121 (30.8)	
LEDD, mg	504.4 ± 257.9	556.0 ± 269.4	455.6 ± 236.7	< 0.001
SES score, %	83.7 ± 13.9	79.3 ± 15.5	87.8 ± 10.6	< 0.001
SES group				
Independent	703 (85.1)	312 (77.4)	391 (92.4)	< 0.001
Dependent	123 (14.9)	91 (22.6)	32 (7.6)	

*Abbreviations*: *EMO* early morning off, *BMI* body mass index, *PD* Parkinson’s disease, *H&Y stage* Hoehn-Yahr stage, *TD* tremor-dominant, *PIGD* Posture instability gait difficulty-dominant, *DA* dopamine agonists, *LEDD* levodopa equivalent daily dosage, *SES* Schwab-England Activities of Daily Living Scale

**Table 2:** Association between baseline characteristics and risk of EMO

Baseline variables	Univariate logistic regression model		Age- and sex-adjusted logistic regression model		Multiple stepwise logistic regression model	
	Odds ratio (95%CI)	*P* value	Odds ratio (95%CI)	*P* value	Odds ratio (95%CI)	*P* value
Age group						
< 55 years	1.00 (reference)		⎼		1.00 (reference)	
55–64 years	1.35 (0.92–2.00)	0.132	⎼	⎼	1.28 (0.78–2.11)	0.323
65–74 years	1.48 (1.00–2.19)	0.050	⎼	⎼	1.79 (1.09–2.96)	0.023
> =75 years	1.28 (0.80–2.06)	0.301	⎼	⎼	1.14 (0.62–2.12)	0.668
Sex						
Male	1.00 (reference)		⎼		⎼	
Female	0.94 (0.73–1.21)	0.634	⎼	⎼	⎼	⎼
BMI groups						
Normal	1.00 (reference)		1.00 (reference)		⎼	⎼
Overweight or obese	0.86 (0.66–1.11)	0.251	0.85 (0.65–1.11)	0.228	⎼	⎼
Education level						
Primary school or lower	1.00 (reference)		1.00 (reference)		⎼	
Middle or high school	1.10 (0.80–1.50)	0.570	1.09 (0.79–1.50)	0.610	⎼	⎼
University or higher	1.03 (0.69–1.53)	0.880	1.04 (0.69–1.56)	0.860	⎼	⎼
Occupation						
Unemployed	1.00 (reference)		1.00 (reference)		⎼	
Worker or farmer	0.70 (0.43–1.12)	0.136	0.66 (0.41–1.07)	0.096	⎼	⎼
Professional technician or others	0.85 (0.53–1.36)	0.508	0.82 (0.51–1.31)	0.399	⎼	⎼
Family history of PD						
No	1.00 (reference)		1.00 (reference)		⎼	⎼
Yes	0.99 (0.64–1.54)	0.964	1.03 (0.66–1.60)	0.908	⎼	⎼
Overall nighttime sleep						
Good	1.00 (reference)		1.00 (reference)		1.00 (reference)	
General	2.17 (1.59–2.97)	< 0.001	2.20 (1.61–3.01)	< 0.001	2.08 (1.41–3.07)	< 0.001
Poor	3.30 (2.36–4.64)	< 0.001	3.34 (2.38–4.71)	< 0.001	3.29 (2.14–5.08)	< 0.001
Nocturnal arousals						
None	1.00 (reference)		1.00 (reference)		⎼	
1–5 times	1.72 (1.08–2.77)	0.024	1.65 (1.04–2.68)	0.038	⎼	⎼
> 5 times	3.17 (1.63–6.27)	0.001	2.98 (1.53–5.95)	0.002	⎼	⎼
PD duration	1.10 (1.06–1.13)	< 0.001	1.10 (1.06–1.14)	< 0.001	1.06 (1.02–1.11)	0.006
H&Y group						
Mild (stage of 1–2)	1.00 (reference)		1.00 (reference)		1.00 (reference)	
Moderate (stage of 2.5–3)	2.75 (2.09–3.64)	< 0.001	2.76 (2.09–3.66)	< 0.001	1.78 (1.24–2.57)	0.002
Severe (stage of 4–5)	3.43 (1.58–8.06)	0.003	3.57 (1.62–8.48)	0.002	1.29 (0.48–3.64)	0.617
Symmetry of symptoms						
No	1.00 (reference)		1.00 (reference)		⎼	
Yes	0.69 (0.41–1.17)	0.174	0.68 (0.40–1.15)	0.158	⎼	⎼
Clinical type						
TD	1.00 (reference)		1.00 (reference)		1.00 (reference)	
PIGD	1.62 (1.18–2.21)	0.003	1.66 (1.22–2.28)	0.002	1.72 (1.16–2.55)	0.007
Mixed	2.02 (1.45–2.80)	< 0.001	2.05 (1.47–2.86)	< 0.001	1.78 (1.17–2.71)	0.007
Medication use						
Levodopa monotherapy	1.00 (reference)		1.00 (reference)		⎼	
Levodopa + DA	1.41 (0.96–2.09)	0.085	1.40 (0.95–2.08)	0.090	⎼	⎼
Others	1.48 (0.98–2.25)	0.064	1.53 (1.01–2.34)	0.046	⎼	⎼
LEDD, mg/100	1.17 (1.11–1.24)	< 0.001	1.17 (1.11–1.25)	< 0.001	1.08 (1.01–1.16)	0.028

*Abbreviations*: *EMO* early morning off, *BMI* body mass index, *PD* Parkinson’s disease, *H&Y stage* Hoehn-Yahr stage, *TD* tremor-dominant, *PIGD* Posture instability gait difficulty-dominant, *DA* dopamine agonists, *LEDD* levodopa equivalent daily dosage

**Fig. 2:**
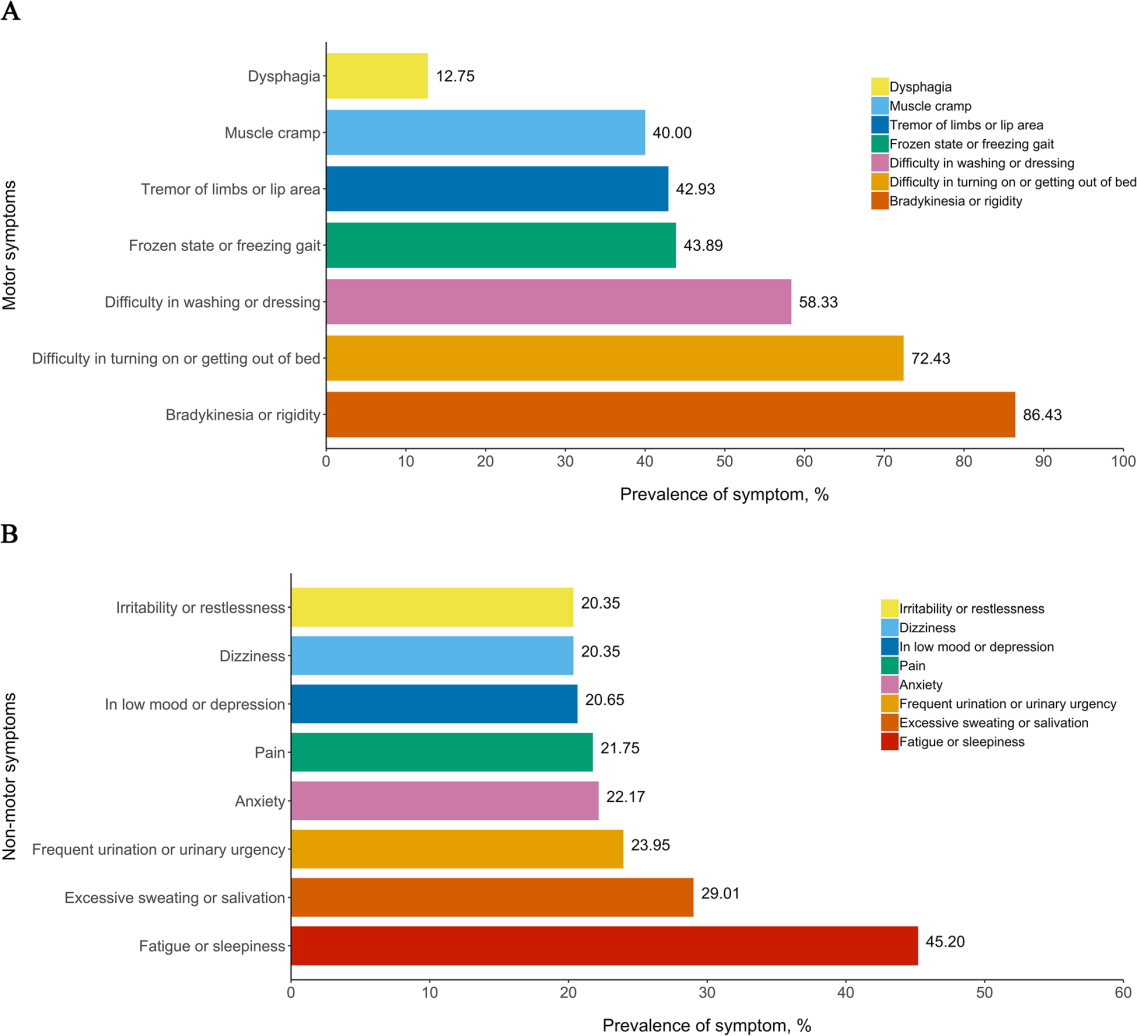
Patterns of motor and non-motor symptoms during EMO

**Fig. 3:**
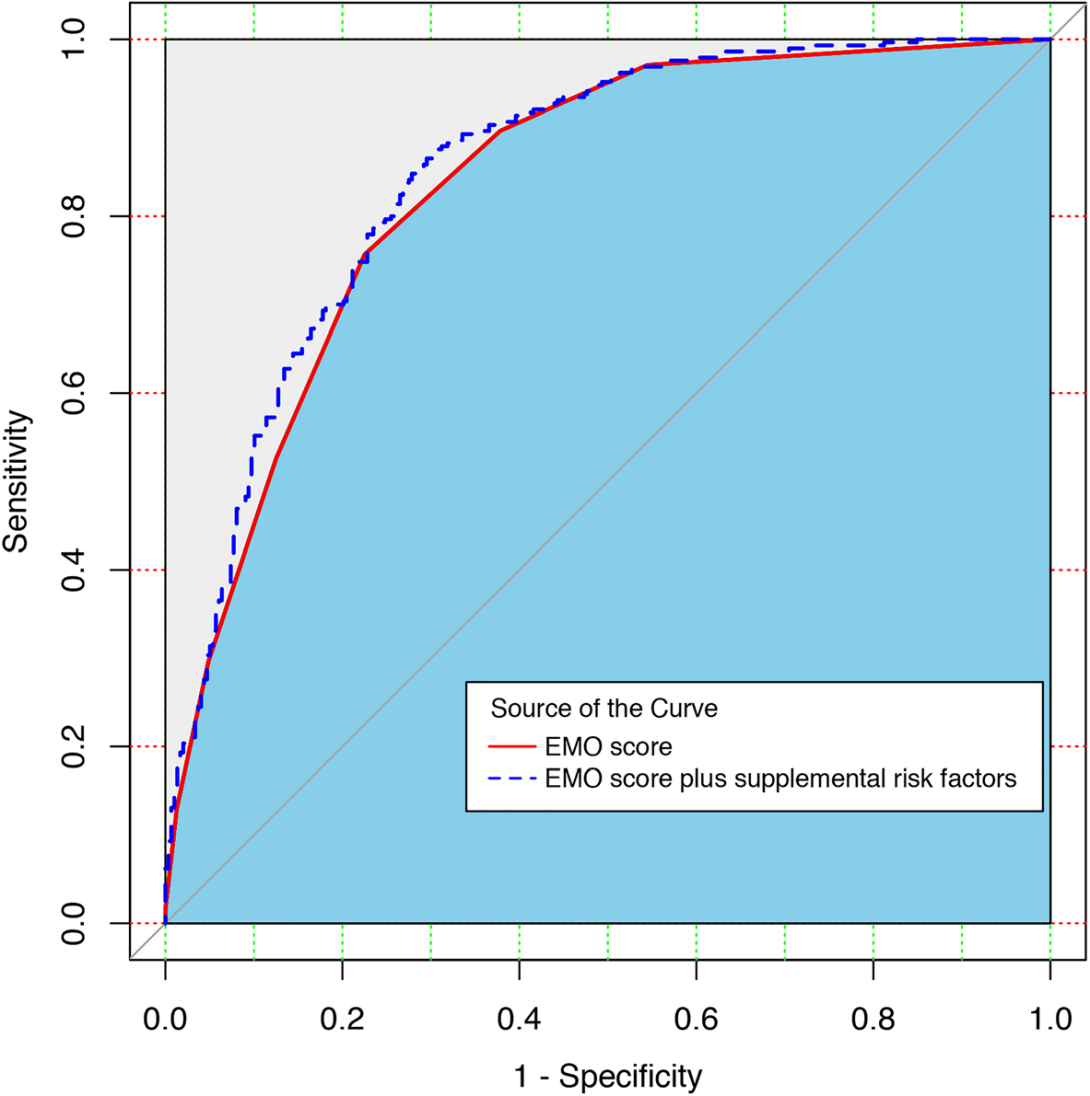
The receiver operating characteristic (ROC) curves of EMO score and supplemental risk factors in predicting probability of EMO in PD patients The red line (solid) and blue line (dotted) illustrates the discriminant properties of EMO score and EMO score plus supplemental risk factors in screening EMO among PD patients, respectively.

**Fig. 4:**
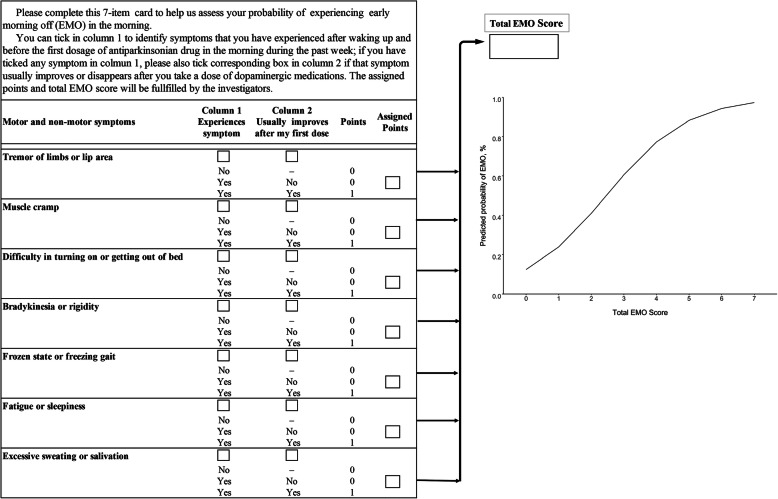
The simple 7-item card for EMO screening in PD patients

The most common MS during EMO was bradykinesia or rigidity (86.4%), difficulty in turning on or getting out of bed (72.4%), and difficulty in washing or dressing (58.3%), while the most common NMS during EMO was fatigue or sleepiness (45.2%), excessive sweating or salivation (29.0%), frequent urination or urinary urgency (24.0%) (Fig. 2). As shown in Table 3, most of these symptoms were also identified as independent predictors of EMO in the multivariate stepwise regression model. The model finally included bradykinesia or rigidity, excessive sweating or salivation, difficulty in turning on or getting out of bed, muscle cramp, fatigue or sleepiness, frozen state or freezing gait, and tremor. We therefore generated an EMO scale comprised of above 7 symptoms with 1 point assigning to each symptom, achieving a total score ranged from 0 to 7. The Youden index was optimized at an EMO score of 2 (J = 0.53). The validation analyses with bootstrapping methodology confirmed a good discrimination of this 7-item scale, yielding a relatively large AUROC of 0.83 (95%CI: 0.80–0.86), a relatively high sensitivity of 75.7% (95%CI: 71.0–89.4%), specificity of 77.5% (95%CI: 62.0–82.6%), PPV of 76.5% (95%CI: 68.8–81.9%), and NPV of 76.7% (95%CI: 71.5–85.8%). As shown in the ROC curves (Fig. 3**)**, the x-axis denotes the false positive rate (1- specificity) while the y-axis denotes the true positive rate (sensitivity). The diagonal line represents the “non-discrimination reference line” which is equivalent to a prediction tool performing not superior to random chance. The red solid line and the blue dotted line represents the discriminative ability of EMO score and EMO score plus aforementioned EMO risk factors, respectively. As expected, a slight but significant performance gain was obtained when adding these supplemental factors into this scale [AUROC: 0.85 (95%CI: 0.80–0.87), *P* = 0.002]. The EMO scale also exhibited a gratifying calibration represented by a Hosmer-Lemeshow statistic of 11.88 (df = 8, *P* = 0.157). Figure 4 illustrates an EMO screening card which can be used as a pragmatic screening tool to establish a patient’s probability of experiencing EMO depending on the status of 7 symptoms.

**Table 3:** Motor and non-motor symptoms significantly associated with EMO in multiple stepwise logistic regression model

Motor or non-motor symptoms	Odds ratio (95%CI)	P value
Tremor		
No	1.00 (reference)	
Yes	1.59 (1.05–2.40)	0.027
Muscle cramp		
No	1.00 (reference)	
Yes	2.00 (1.29–3.14)	0.002
Difficulty in turning on or getting out of bed		
No	1.00 (reference)	
Yes	2.05 (1.36–3.10)	0.001
Bradykinesia or rigidity		
No	1.00 (reference)	
Yes	4.97 (3.17–7.87)	< 0.001
Frozen state or freezing gait		
No	1.00 (reference)	
Yes	1.62 (1.04–2.54)	0.032
Fatigue or sleepiness		
No	1.00 (reference)	
Yes	1.99 (1.29–3.07)	0.002
Excessive sweating or salivation		
No	1.00 (reference)	
Yes	2.25 (1.35–3.81)	0.002

We also identified eight statistically significant determinants associated with daily functional dependency as defined by SES: age group, sex, PD duration, education level, clinical type, H&Y stage, overall nighttime sleep, and EMO state (Table S3). The multivariable-adjusted odds of functional dependency were 87% higher (OR = 1.87, 95%CI: 1.07–3.32) for EMO patients compared with their counterparts, proving an independent negative impact of EMO on PD patient’s QOL.

## Discussion

EMO is such a common phenomenon to PD patients across all disease stages at a high prevalence of 49.2%. Nonideal nighttime sleep, advanced H&Y stages, long PD duration, PIGD or mixed subtypes, and high levodopa dose were proved to be independently associated with higher EMO risk. To our knowledge, there was no validated simple tools to exclusively distinguish EMO sufferers from PD patients, as the case with 9-symptom Wearing-off Questionnaire (WOQ-9) to identify wearing-off, as yet. The only existing EMO-related screening tool, the Time-to-On questionnaire (TOQ), yet was mainly targeted at documenting “delayed ON” phenomenon and related symptoms in turning ON among already confirmed EMO patients, and was relatively complicated [16]. In this context, our study has put forward an easy-to-use 7-item self-assessment instrument that can be used to screen for EMO in clinical practice for the first time. Besides, we have identified EMO as an independent risk factor predisposing to functional dependency in daily living.

Existing evidence investigating wearing-off state of PD patients mainly focused on daytime manifestation, while nighttime and early-morning symptoms attracted far less attention [6, 13–16, 27–30]. Likewise, although wearing-off has been a well-acknowledged concept for neurologists among their routine care for PD patients, EMO was usually neglected or underestimated, which might lead to unconscious hazard, insufficient management and increased care burden [14, 15]. In our study, EMO was observed in almost half of the unselected PD patients with a wide range of motor and no-motor fluctuations. In particular, even more than one third of those at early stage were also experiencing EMO, and this frequency increased remarkably with the exacerbation of PD. These findings were in line with the observations of Rizos et al. [14] who reported a total prevalence as high as 59.7% and a subgroup rate of 44.3% for mild ones among 320 PD outpatients from several European clinical centers. They also depicted some common NMS, nearly consistent with ours, including fatigue, urinary urgency, dribbling of saliva, anxiety, low mood, pain, etc. Prior to this study, an estimated 58.5% of early morning akinesia and 79.8% of poor bodily movements at wake was once reported in a United Kingdom study [23] and a Japanese nationwide survey [15], respectively. Most recently, another single-center pilot study [16] conducted at Roma among a relatively small sample size of 151 Caucasian PD patients also found 64.2% of PD patients experiencing prolonged morning OFF periods in clinical practice. Similarly, the most common symptoms in waiting to turn ON were slowness and fatigue as well. Variations in the exact occurrence rate was partially due to heterogeneity in sampled populations and examine methods used in various studies. Whereas, EMO was convinced to be highly prevalent across diverse ethnic populations. In agreement with Rizos et al. [14], we further found that the baseline characteristics of EMO positive patients and negative patients matched closely in terms of most demographic characteristics, but differed noticeably in nighttime sleep quality, PD duration, H&Y stages, clinical subtypes, LEDD, and all the motor/non-motor dysfunctions. As the only modifiable risk factor listed above, improving nighttime sleep should attract substantial attention in the preliminary prevention of EMO among PD patients [31].

A previous study by Stacy [6] has pointed out the necessity of developing tools to accurately grasp patients’ subjective symptoms of wearing-off, which applies equally to EMO as emphasized by other specialists [14]. Considering that some MS and NMS are exclusive to the morning period and don’t reoccur throughout the daytime, an EMO-specific screening scale is warranted. In this regard, we have deliberately selected seven most dominant symptoms associated with EMO through fitting a stepwise regression model and proved that the resulting 7-item scale achieved a high accuracy of 83.5%. Except for four MS consistent with WOQ-9, two dysautonomic NMS (fatigue and sleepiness, excessive sweating or salivation) as well as difficulty in turning on or getting out of bed, appeared to be more specific in recognizing EMO. As far as we know, this is the first promising tool to detect EMO from PD patients with reasonably good discrimination and calibration up to date. Other advantages of our scale included that it can be quickly completed by patients themselves prior to a doctor’s interview, thus saves consultation time and alleviates clinician’s workload. It can be deemed as a shorter version of the TOQ tool as well. Another medical implication is that this scale might play an important role in raising EMO awareness, prescreening suspected patients, and assisting clinician’s decision-making. It could assist healthcare professionals in deciding which patients might benefit from additional treatment and therefore undertaking timely targeted treatment adjustment according to personal clinical phenotypes.

It has been confirmed that EMO has substantial impact on PD patients’ QOL and caregiver burden [14, 15, 23]. One possible reason was EMO-induced dystonia and consequent loss of independency in daily living, as shown in our results [32]. Based on robust evidence from randomized controlled clinical trials, a number of medications have been proved to be effective adjuncts to levodopa treatment in managing wearing-off, such as MAO-B inhibitors (e.g. rasagiline), DA (e.g. pramipexole, ropinirole), and catechol-*O*-methyltransferase (COMT) inhibitors (e.g. entacapone) etc. [29, 30, 33] The current state of managing EMO is also promising with increasingly numerous pharmacologic therapies aimed at improving nocturnal sleep disorders and alleviating early-morning motor dysfunction. Potential treatment options included but were not limited to long-acting rotigotine, rasagiline, cabergoline, extended-release pramipexole, apomorphine, and orally inhaled levodopa [17–21, 24, 25, 34]. Our study was not powerful for, or focusing on, comparing the therapeutic effect of various antiparkinsonian medicines, thus failed to detect any significant differences between different therapy regimens. Nevertheless, we have revealed a causative role for levodopa dose in EMO risk as suggested by other randomized trials and cohort studies in predicting wearing off or motor complications [8, 13].

Several strengths of our study deserve mention. Firstly, this is a nationwide multicenter study which recruited a representative sample from real world situation with limited referral bias. Stringent quality control procedures were employed during information collection and data analyses, including unified training of all investigators, standardized protocols, and double-check of analyzed data. It is also, to our knowledge, the largest and most comprehensive study on EMO in view of the assessment of a wide range of variables. Notably, we specifically measured nighttime sleep quality and excluded the effect of poor sleep pattern on early morning state. Yet, these results should also be interpreted in light of some limitations. Firstly, all the MS and NMS were collected via a retrospective questionnaire, which was probably subject to recall bias. However, each symptom was ascertained only if it was improved after a morning dopaminergic treatment, thus making sure that it reflected a true symptom fluctuation over EMO period. Secondly, as there was so far no consensus definition of EMO, in this study the EMO patients were pre-identified by experienced specialists using empiric diagnostic criteria. Some subtle signs of EMO, especially non-motor fluctuations, might be easily missed in a short clinical visit. This probably resulted in an underestimation of EMO prevalence and some misclassification bias. However, it was not our intention to put forward a diagnostic instrument, but rather, a screening tool to facilitate the timely recognition of EMO in clinical practice. From this perspective, this 7-item scale should be deemed as a small first step in the investigation of EMO. Further validation and refinement of this scale, as well as evidence to determine whether its routine use is of clinical value in identifying EMO patients and enhancing patients care are warranted. Besides, considering the intrinsic variations in genetic and environmental background across different ethnicities, inclusion of Chinese patients only might limit the generalizability of our findings to other ethnical populations. Therefore, this novel scale awaits replication and external confirmation in other populations as well.

## Conclusions

In this study, we highlighted the grim situation of EMO prevalence among Chinese PD patients and derived an EMO-specific screening scale with advantages of simple implementation, little time-consuming, and high accuracy. Given that EMO patients generally confer a higher risk of being functional dependent in daily life, it’s necessary to increase EMO awareness and promptly take effective strategies to control it. Following successful external validation, this EMO scale is expected to facilitate earlier detection of EMO, allow timely therapy modification, enable optimal symptomatic control, thereby contribute to all-day management and QOL improvement.

## Supplementary information

**Additional file 1 TableS1**. Patient questionnaire on common motor and non-motor symptoms.

**Additional file 2 TableS2**. Motor and non-motor symptoms of the study participants.

**Additional file 3 TableS3**. Baseline variables significantly associated with dependency of daily living in multiple stepwise logistic regression model.

**Additional file 4 TableS4**. Investigators of the China Early Morning Off Study Group.

## Data Availability

All the data mentioned in this article are available on published article.

## Abbreviations

AUROC: Area under the receiver operative characteristic curve
AIC: Akaike information criteria
BMI: Body mass index
CIs: Confidence intervals
COMT: Catechol-*O*-methyltransferase inhibitors
DA: Dopamine agonists
EMO: Early morning off
H&Y: Hoehn-Yahr
J: Youden index
MAO-B: Monoamine oxidase type B
LEDD: Levodopa equivalent daily dosage
MS: Motor symptoms
MMSE: Mini-Mental State Examination
MDS-UPDRS: Movement Disorders Society Unified Parkinson Disease Rating Scale
NPV: Negative predictive value
NMS: Non-motor symptoms
NMSQ-30: 30-item NMS questionnaire
ORs: Odds ratios
PD: Parkinson’s disease
PPV: Positive predictive value
PIGD: Posture instability gait difficulty-dominant
QOL: Quality of life
SES: Schwab-England Activities of Daily Living Scale
SD: Standard deviation
TD: Tremor-dominant
WOQ-9: 9-symptom Wearing-off Questionnaire
