# Supplementary material for: Early morning off in patients with Parkinson’s disease: a Chinese nationwide study and a 7-question screening scale
Source: Transl Neurodegener. 2020 Jul 6;9:29. doi: 10.1186/s40035-020-00208-z (PMC7336490; doi:10.1186/s40035-020-00208-z)
Supplement: Supplementary file 1 — Additional file 1 TableS1. Patient questionnaire on common motor and non-motor symptoms. [file 40035_2020_208_MOESM1_ESM.docx]

**Supplemental Table 1. Patient questionnaire on common motor and non-motor symptoms.**

| Please complete this questionnaire to help us understand if you have experienced any of these motor and non-motor symptoms after waking up and before the first dosage of antiparkinsonian drug in the morning during the past week.  You can tick in column 1 to identify symptoms that you have experienced after waking up and before the first dosage of antiparkinsonian drug in the morning during the past week. Please tick the box “Yes” if you have experienced it, if not tick the “No” box.  If you have ticked “Yes” in any symptom of column 1, please also tick corresponding box in column 2 to clarify whether this symptom usually improves or disappears after you take a dose of dopaminergic medications. Please tick the box “Yes” if it improves after medication use, if not tick the “No” box. | | | | | |
| --- | --- | --- | --- | --- | --- |
|  | | Column 1  Experiences  symptom | | Column 2  Usually improves  after my first dose | |
|  | | Yes | No | Yes | No |
| 1 | Tremor of limbs or lip area |  |  |  |  |
| 2 | Muscle cramp |  |  |  |  |
| 3 | Difficulty in turning on or getting out of bed |  |  |  |  |
| 4 | Bradykinesia or rigidity |  |  |  |  |
| 5 | Frozen state or freezing gait |  |  |  |  |
| 6 | Dysphagia |  |  |  |  |
| 7 | Difficulty in washing or dressing |  |  |  |  |
| 8 | Pain that affects sleep posture (upper or lower limbs) forces awake early in the morning |  |  |  |  |
| 9 | Fatigue or sleepiness |  |  |  |  |
| 10 | Nocturnal frequent urination or urinary urgency |  |  |  |  |
| 11 | In low mood or depression |  |  |  |  |
| 12 | Excessive sweating or salivation |  |  |  |  |
| 13 | Dizziness |  |  |  |  |
| 14 | Irritability or restlessness |  |  |  |  |
| 15 | Anxiety |  |  |  |  |
