# Supplementary material for: Early morning off in patients with Parkinson’s disease: a Chinese nationwide study and a 7-question screening scale
Source: Transl Neurodegener. 2020 Jul 6;9:29. doi: 10.1186/s40035-020-00208-z (PMC7336490; doi:10.1186/s40035-020-00208-z)
Supplement: Supplementary file 2 — Additional file 2 TableS2. Motor and non-motor symptoms of the study participants. [file 40035_2020_208_MOESM2_ESM.docx]

**Supplemental Table 2. Motor and non-motor symptoms of the study participants.**

| Symptoms | Overall  (n=942) | EMO patients (n=463) | Non-EMO patients (n=479) | P Value |
| --- | --- | --- | --- | --- |
| Tremor of limbs or lip area |  |  |  |  |
| No | 562 (68.9) | 226 (57.1) | 336 (80.0) | <0.001 |
| Yes | 254 (31.1) | 170 (42.9) | 84 (20.0) |  |
| Muscle cramp |  |  |  |  |
| No | 608 (74.3) | 240 (60.0) | 368 (88.0) | <0.001 |
| Yes | 210 (25.7) | 160 (40.0) | 50 (12.0) |  |
| Difficulty in turning on or getting out of bed |  |  |  |  |
| No | 406 (49.8) | 110 (27.6) | 296 (71.2) | <0.001 |
| Yes | 409 (50.2) | 289 (72.4) | 120 (28.8) |  |
| Bradykinesia or rigidity |  |  |  |  |
| No | 319 (39.3) | 54 (13.6) | 265 (64.2) | <0.001 |
| Yes | 492 (60.7) | 344 (86.4) | 148 (35.8) |  |
| Frozen state or freezing gait |  |  |  |  |
| No | 586 (71.6) | 225 (56.1) | 361 (86.4) | <0.001 |
| Yes | 233 (28.4) | 176 (43.9) | 57 (13.6) |  |
| Dysphagia |  |  |  |  |
| No | 745 (91.3) | 349 (87.2) | 396 (95.2) | <0.001 |
| Yes | 71 (8.7) | 51 (12.8) | 20 (4.8) |  |
| Difficulty in washing or dressing |  |  |  |  |
| No | 456 (56.5) | 165 (41.7) | 291 (70.8) | <0.001 |
| Yes | 351 (43.5) | 231 (58.3) | 120 (29.2) |  |
| Pain |  |  |  |  |
| No | 697 (85.3) | 313 (78.2) | 384 (92.1) | <0.001 |
| Yes | 120 (14.7) | 87 (21.8) | 33 (7.9) |  |
| Fatigue or sleepiness |  |  |  |  |
| No | 577 (71.2) | 217 (54.8) | 360 (87.0) | <0.001 |
| Yes | 233 (28.8) | 179 (45.2) | 54 (13.0) |  |
| Frequent urination or urinary urgency |  |  |  |  |
| No | 642 (82.4) | 289 (76.1) | 353 (88.5) | <0.001 |
| Yes | 137 (17.6) | 91 (23.9) | 46 (11.5) |  |
| In low mood or depression |  |  |  |  |
| No | 693 (85.9) | 315 (79.3) | 378 (92.2) | <0.001 |
| Yes | 114 (14.1) | 82 (20.7) | 32 (7.8) |  |
| Excessive sweating or salivation |  |  |  |  |
| No | 651 (80.3) | 279 (71.0) | 372 (89.0) | <0.001 |
| Yes | 160 (19.7) | 114 (29.0) | 46 (11.0) |  |
| Dizziness |  |  |  |  |
| No | 702 (86.1) | 317 (79.6) | 385 (92.3) | <0.001 |
| Yes | 113 (13.9) | 81 (20.4) | 32 (7.7) |  |
| Irritability or restlessness |  |  |  |  |
| No | 705 (86.3) | 317 (79.6) | 388 (92.6) | <0.001 |
| Yes | 112 (13.7) | 81 (20.4) | 31 (7.4) |  |
| Anxiety |  |  |  |  |
| No | 694 (85.0) | 309 (77.8) | 385 (91.9) | <0.001 |
| Yes | 122 (15.0) | 88 (22.2) | 34 (8.1) |  |

Abbreviations: EMO, early morning off.
