# Supplementary material for: Early morning off in patients with Parkinson’s disease: a Chinese nationwide study and a 7-question screening scale
Source: Transl Neurodegener. 2020 Jul 6;9:29. doi: 10.1186/s40035-020-00208-z (PMC7336490; doi:10.1186/s40035-020-00208-z)
Supplement: Supplementary file 3 — Additional file 3 TableS3. Baseline variables significantly associated with dependency of daily living in multiple stepwise logistic regression model. [file 40035_2020_208_MOESM3_ESM.docx]

**Supplemental Table 3. Baseline variables significantly associated with dependency of daily living in multiple stepwise logistic regression model.**

| Baseline variables | Odds ratio (95%CI) | P value |
| --- | --- | --- |
| Age group |  |  |
| <55 years | 1.00 (reference) |  |
| 55-64 years | 1.28 (0.54-3.27) | 0.587 |
| 65-74 years | 1.42 (0.62-3.57) | 0.430 |
| >=75 years | 3.08 (1.19-8.51) | 0.024 |
| Sex |  |  |
| Male | 1.00 (reference) |  |
| Female | 0.53 (0.31-0.90) | 0.020 |
| PD duration | 1.09 (1.03-1.15) | 0.004 |
| Education level |  |  |
| Primary school or lower | 1.00 (reference) |  |
| Middle or high school | 0.64 (0.35-1.16) | 0.138 |
| University or higher | 0.40 (0.17-0.88) | 0.026 |
| Clinical type |  |  |
| TD | 1.00 (reference) |  |
| PIGD | 2.10 (1.14-3.94) | 0.019 |
| Mixed | 1.52 (0.79-2.92) | 0.211 |
| H&Y stages | 7.26 (4.60-12.01) | <0.001 |
| Overall nighttime sleep |  |  |
| Good | 1.00 (reference) |  |
| General | 1.10 (0.57-2.17) | 0.771 |
| Poor | 2.29 (1.20-4.45) | 0.013 |
| EMO state |  |  |
| No | 1.00 (reference) |  |
| Yes | 1.87 (1.07-3.32) | 0.031 |

Abbreviations: PD, Parkinson’s disease; TD, tremor-dominant; PIGD, Posture instability gait difficulty-dominant; H&Y stage, Hoehn-Yahr stage; EMO, early morning off.
