# Supplementary material for: Early morning off in patients with Parkinson’s disease: a Chinese nationwide study and a 7-question screening scale
Source: Transl Neurodegener. 2020 Jul 6;9:29. doi: 10.1186/s40035-020-00208-z (PMC7336490; doi:10.1186/s40035-020-00208-z)
Supplement: Supplementary file 4 — Additional file 4 TableS4. Investigators of the China Early Morning Off Study Group. [file 40035_2020_208_MOESM4_ESM.docx]

**Supplemental Table 4. Investigators of the China Early Morning Off (EMO) Study Group.**

| ID | Investigator | Affiliation | Location |
| --- | --- | --- | --- |
| 1 | Wei Mao | Department of Neurobiology, Xuanwu Hospital of Capital Medical University | Beijing, Northern China |
| 2 | Tao Wang | Department of Neurology, Union Hospital, Tongji Medical College, Huazhong University of Science and Technology | Wuhan, Hubei, Central China |
| 3 | Tao Feng | Beijing Tiantan Hospital, Capital Medical University | Beijing, Northern China |
| 4 | Hongmei Yu | Department of Neurology, First Affiliated Hospital of China Medical University | Shenyang, Liaoning, Northeast China |
| 5 | Huifang Shang | Department of Neurology, West China Hospital, Sichuan University | Chengdu, Sichuan, Southwest China |
| 6 | Han Wang | Department of Neurology, Peking Union Medical College Hospital | Beijing, Northern China |
| 7 | Chun-Feng Liu | Department of Neurology, the Second Affiliated Hospital of Soochow University | Suzhou, Jiangsu, Eastern China |
| 8 | Min Ye | Department of Neurology, the Affiliated BenQ Hospital of Nanjing Medical University | Nanjing, Jiangsu, Eastern China |
| 9 | Jifeng Guo | Department of Neurology, Xiangya Hospital, Central South University | Changsha, Hunan, Central China |
| 10 | Lei Chen | Department of Neurology, Tianjin Huan Hu Hospital, Tianjin Key Laboratory of Cerebrovascular and Neurodegenerative Disease | Tianjin, Northern China |
| 11 | Ping Liu | The Sixth Medical Center of PLA General Hospital | Beijing, Northern China |
| 12 | Oumei Cheng | Department of Neurology, the First Affiliated Hospital, Chongqing Medical University | Chongqing, Southwest China |
| 13 | Rong hua Dou | Cangzhou Hospital of Integrated TCM-WM·HEBEI | Guangzhou, Guangdong, Southern China |
| 14 | Keping Yang | The Second People's Hospital of Guiyang | Guiyang, Guizhou, Southwest China |
| 15 | Qiaoyun Dong | Cangzhou Central Hospital | Guangzhou, Guangdong, Southern China |
| 16 | Limei Zhang | The 2nd Affiliated Hospital of Harbin Medical University | Harbin, Heilongjiang, Northeast China |
| 17 | Suzhi Liu | Taizhou Hospital of Zhejiang | Taizhou, Zhejiang, Eastern China |
| 18 | Rong Zhang | Zhangjiagang TCM Hospital Affiliated to Nanjing University of Chinese Medicine | Zhangjiagang, Jiangsu, Eastern China |
| 19 | Lifang Zhang | General Hospital of Ningxia Medical University | Changsha, Hunan, Central China |
| 20 | Ying Chang | China-Japan Union Hospital, Jilin University | Changchun, Jilin, Northeast China |
| 21 | Xiaohua Xu | The Fifth Affiliated Hospital of Sun Yat-sen University | Guangzhou, Guangdong, Southern China |
| 22 | Li Kou | The Fifth Affiliated Hospital of Sun Yat-sen University | Guangzhou, Guangdong, Southern China |
| 23 | Liping Cheng | Handan NO.1 Hospital | Handan, Hebei, Northern China |
| 24 | Meiji Chang | Changchun Central Hospital | Changchun, Jilin, Northeast China |
| 25 | Yong You | The Second Affiliated Hospital of Hainan Medical University | Haikou, Hainan, Southern China |
| 26 | Cui Wang | Dalian Municipal Central Hospital | Dalian, Liaoning, Northeast China |
| 27 | Jianjun Lu | Guangdong Second Provincial General Hospital | Guangzhou, Guangdong, Southern China |
| 28 | Mengyan Li | Guangzhou First People's Hospital | Guangzhou, Guangdong, Southern China |
| 29 | Yan Chen | Shandong Provincial Hospital affiliated to Shandong University | Jinan, Shandong, Northern China |
| 30 | Yongqiu Wang | Ningbo Medical Center Lihuili Hospital | Ningbo, Zhejiang, Eastern China |
| 31 | Cuiping Zhao | Qilu Hospital of Shandong University | Jinan, Shandong, Northern China |
| 32 | Jin Li | Wuhan Brain Hospital | Wuhan, Hubei, Central China |
| 33 | Zhongwen Zhang | Wuhan No.1 Hospital, Wuhan, Hubei, China | Wuhan, Hubei, Central China |
| 34 | Ting Zhou | Wuhan Union Dongxihu Hospital | Wuhan, Hubei, Central China |
| 35 | Hong Liu | Heping Hospital Affiliated to Changzhi Medical College | Changzhi, Shanxi, Northern China |
| 36 | Xiaohong Li | Dalian Municipal Friendship Hospital | Dalian, Liaoning, Northeast China |
| 37 | Shunliang Xu | The Second Hospital of Shandong University | Jinan, Shandong, Northern China |
| 38 | Hongbo Xiao | Anshan Central Hospital | Anshan, Liaoning, Northeast China |
| 39 | Qiang Sun | Taihe Hospital, Hubei University of Medicine | Wuhan, Hubei, Central China |
| 40 | Junwu Zhao | Weihai Municipal Hospital | Wuhan, Hubei, Central China |
| 41 | Lei Wu | The First Affiliated Hospital of University of Science and Technology of China | Hefei, Anhui, Eastern China |
| 42 | Jiaying Xu | People's Hospital of Tiantai | Taizhou, Zhejiang, Eastern China |
| 43 | Lijun Guo | Affiliated Hospital of Xiangnan University | Chunzhou, Hunan, Central China |
| 44 | Yamian Gao | Hejian People's Hospital | Cangzhou, Hebei, Northern China |
| 45 | Yan Yang | Affiliated Hospital of Jining Medical University | Jining, Shandong, Northern China |
| 46 | Cuihong Ma | Chengde Central Hospital | Chengde, Hebei, Northern China |
| 47 | Lu Shi | Ansteel Group Hospital | Anshan, Liaoning, Northeast China |
| 48 | Xiangqing Li | Zibo Central Hospital | Zibo, Shandong, Northern China |
| 49 | Xiaofan Yang | Hongqi Affiliated Hospital of Mudanjiang Medical College | Mudanjiang, Heilongjiang, Northeast China |
| 50 | Qingzhen Zhou | Affliated Dongfeng Hospital, Hubei University of Medicine | Wuhan, Hubei, Central China |
| 51 | Shuang Liu | Jinan Central Hospital Affiliated to Shandong University | Jinan, Shandong, Northern China |
| 52 | Zhanhua Liang | The First Affiliated Hospital of Dalian Medical University | Dalian, Liaoning, Northeast China |
| 53 | Juping He | Zhejiang Dongyang People's Hospital | Wenzhou, Zhejiang, Eastern China |
| 54 | Weihua Jia | Shijingshan Teaching Hospital, The Capital Medical University | Beijing, Northern China |
| 55 | Xiaofeng Lei | Tianjin 4th Center Hospital | Tianjin, Northern China |
